# Gut Microbiological Disorders Reduce Semen Utilization Rate in Duroc Boars

**DOI:** 10.3389/fmicb.2020.581926

**Published:** 2020-10-08

**Authors:** Liangliang Guo, Yinghui Wu, Chao Wang, Hongkui Wei, Jiajian Tan, Haiqing Sun, Siwen Jiang, Jian Peng

**Affiliations:** ^1^Department of Animal Nutrition and Feed Science, College of Animal Science and Technology, Huazhong Agricultural University, Wuhan, China; ^2^YangXiang Joint Stock Company, Guigang, China; ^3^Key Lab of Agricultural Animal Genetics, Breeding and Reproduction of Ministry of Education and Key Lab of Swine Genetics and Breeding of Ministry of Agriculture, Huazhong Agricultural University, Wuhan, China; ^4^The Cooperative Innovation Center for Sustainable Pig Production, Wuhan, China

**Keywords:** boar, semen utilization rate, gut microbiota, intestinal permeability, inflammatory status

## Abstract

Although rising evidence suggests that the gut microbiota is closely related to host health, the effects of gut microbiota on male fertility are still rarely explored. This study was to investigate the gut microbiota composition and function, fecal short-chain fatty acids (SCFA), intestinal permeability, and systemic inflammatory status of Duroc boar with high (H group, 100%) and low (L group, <80%) semen utilization rate. Fecal samples, analyzed by 16S ribosomal RNA gene sequencing, displayed taxonomic and functional changes between boars with high and low semen utilization rates. For the gut microbiota composition of the boars, four genera were different between the two groups. The [*Ruminococcus*] and *Sphingobium* were enriched in L group boars, then negatively correlated with the semen utilization rate. While *RFN20* and *Paludibacter* were enhanced in the H group, only *RFN20* showed a significantly positive correlation with the semen utilization rate of boars. In addition, changes in the metabolic function of the gut microbiota of the two groups were found, including altered branched-chain fatty acid (BCFA) production. Significant increases in plasma endotoxin, zonulin, diamine oxidase, and lipocalin-2 levels were observed in boars with low semen utilization, and also, a similar trend in IL-6 and TNF-α was found. However, the concentration of IL-10 in plasma of boars with high semen utilization rate showed an increasing tendency. These results indicated increased intestinal permeability and systemic inflammation in boars with low semen utilization. Data showed that the composition and functions of gut microbiota varied between boars with high or low semen utilization rates, while the semen utilization rate is notably correlated with the gut microbiota composition, intestinal permeability, and inflammatory status of the boar.

## Introduction

Male fertility is of paramount importance for breeding animal herds when artificial insemination is involved (Waberski et al., 2008). Therefore, monitoring and analyzing the quality of boar semen has vast economic meaning for the pig industry (Smital, 2009). Many factors including varicocele, accessory gland infection, immunological factors, and endocrine causes can adversely affect sperm quality (Omu, 2013). Being one of the largest immune, endocrine, and neural organs in the body, the gut may affect male fertility, which has been underestimated. Researches showed that the gut microbiome influences host endocrine and immune functions (Smith et al., 2013; Rastelli et al., 2019). Moreover, emerging data have demonstrated that an aberrant gut microbiota composition is associated with several diseases, including metabolic disorders and inflammatory bowel disorder (Koh et al., 2016). However, the exact effects of gut microbiota on reproduction have not been elucidated.

Healthy gut microbiota plays many beneficial roles in the host gut, such as resistance to colonization of pathogens, immune regulation, and assisted digestion. Simultaneous production of bacterial metabolites, such as short-chain fatty acids (SCFA) and lactic acid, affects the health of the host (Sekirov et al., 2010; Flint et al., 2012), while there is new evidence that dysregulation of gut microbiota can lead to increased intestinal permeability and may act as a part in the development of chronic low-grade inflammation in the host (Cani et al., 2007; Yu et al., 2016). This chronic inflammation may be related to the gut microbiota components like lipopolysaccharide (LPS), which is a chronic innate immune stimulator in pigs (Webel et al., 1997; Gabler et al., 2008; Weber and Kerr, 2008). Once LPS enters the blood circulation through the gut barrier, it may bind to reproductive tract cells or indirectly stimulate immune cells, thereby affecting steroidogenesis and sperm quality (Hedger, 2011).

In this study, we evaluated the compositional, functional, and metabolic differences in the gut microbiota between the Duroc boars with high (*n* = 29) or low (*n* = 11) semen utilization rates. We also attempted to investigate the intestinal permeability and inflammatory status of boars of different semen utilization rates. The discoveries will provide new insights into increasing semen quality of the boar during high-intensity production and establish a foundation for further research on the impact of gut microbiota on male reproduction.

## Materials and Methods

### Animals

A total of 40 Duroc boars aged from12 to 54 months were selected in an artificial insemination center of Yangxiang Joint Stock Company (Guangxi, China). Boar feeding conditions were as our previous study (Wu et al., 2019a). Semen samples were collected by gloved-hand techniques. After collecting, four semen parameters were assessed, including semen volume, sperm concentration, sperm motility, and abnormal sperm rate, according to the method we described earlier (Wu et al., 2019b). According to the cut-off value recommended by previous studies, boar semen with sperm motility below 70% or abnormal rate above 20% is considered unusable for artificial insemination (Rodriguez et al., 2013; Wu et al., 2019a). Since the quality of the semen collected in a single shot is difficult to represent the real situation of the boar, we introduced a new concept called semen utilization rate, which was calculated based on the semen quality of each ejaculation within 3 months (the number of times that semen can be used/the total number of times semen collected). In the experiment, 40 boars were divided separately into a high semen utilization rate group and a low semen utilization rate group. The 11 boars with a semen utilization rate <80% were defined as “low semen utilization rate group” (L group), and those with a 100% semen utilization rate were defined as “high semen utilization rate group” (H group; Table 1). All animal research procedures were conducted in accordance with animal research guidelines issued by the Institutional Animal Care and Use Committee of Huazhong Agricultural University (permit number HZAUSW-2018-014).

**Table 1:** Semen quality parameters of Duroc boars in different semen utilization rate groups (*n* = 40).

	Low utilization rate group(<80%, *n* = 11)	High utilization rate group (100%, *n* = 29)	*p*-value^*^
Semen volume, ml	108.98 ± 26.35^b^	135.23 ± 37.04^a^	0.038
Sperm concentration, ×10^7^ ml^−1^	61.53 ± 23.32	54.72 ± 16.52	0.307
Total sperm number, ×10^9^/ejaculate	63.10 ± 14.70	70.44 ± 17.11	0.217
Functional sperm number, ×10^9^/ejaculate	45.03 ± 12.99^B^	59.89 ± 15.24^A^	0.007
Sperm motility, %	86.4 ± 4.54^B^	91.14 ± 2.08^A^	<0.001
Abnormal sperm rate, %	18.3 ± 4.34^A^	6.90 ± 1.92^B^	<0.001
Semen utilization, %	58.35 ± 0.16^B^	100^A^	<0.001

The results are presented as mean ± SD.

* Different superscript letters in the same row indicate significant differences.

^A,B^*p* < 0.01; ^a,b^ *p* < 0.05.

### Sample Collections

Blood samples were harvested by venipuncture from the hindlimb vein of boars in ejaculations. The blood sample was then centrifuged at 3,000 × *g* for 10 min at 4°C to obtain a plasma sample and stored at −80°C until analysis. The boar’s rectum was massaged to stimulate boar defecation, and then fresh feces were collected and placed in a sterile 5-ml cryopreservation tube. Stool samples were put on the ice first and then transfer it to −80°C for subsequent analysis.

### Detection of Intestinal Permeability and Systemic Inflammation Markers in Plasma

Zonulin is a medium well known to modulate gut permeability by modifying intracellular tight junctions. Increased circulating zonulin is considered a potential sign of increased intestinal permeability (Maria Moreno-Navarrete et al., 2012). Simultaneously, endotoxin and diamine oxidase in plasma are also vital indicators for intestinal epithelial permeability and integrity (Luk et al., 1983). The plasma zonulin, endotoxin, diamine oxidase, interleukin-6 (IL-6), IL-10, tumor necrosis factor (TNF)-α, and total lipocalin-2 concentrations were determined by using porcine enzyme-linked immunosorbent assay kits (mlbio, Shanghai, China), according to the manufacturer’s instructions.

### Examination of Fecal Short-Chain Fatty Acids and Lactic Acid

The SCFA (acetate, propionate, butyrate, and valerate) concentrations and branched-chain fatty (BCFA; isobutyrate and isovalerate) concentrations in feces of the boars were analyzed by a gas chromatographic method, as described by our previous study (Cheng et al., 2019). Fecal lactic acid concentrations were determined by using porcine enzyme-linked immunosorbent assay kits (mlbio, Shanghai, China), according to the manufacturer’s instructions.

### 16S rRNA Gene Sequencing and Data Processing

Total genomic DNA was extracted from each fecal sample using QIAamp Fast DNA stool Minikit (Qiagen, Germany), according to the manufacturer’s instructions. The forward primer 341F (5'-ACT CCT ACG GGA GGC AGC AG-3') and the reverse primer 806R (5'-GGA CTA CHV GGG TWT CTA AT-3') were used for amplification of the V3-V4 hypervariable region of a 16S rRNA gene. Thirty cycles of PCR were performed at an annealing temperature of 56°C. PCR amplicons were purified and quantified. The PCR products were used for the construction of the libraries and then paired-end sequenced (2 × 250) on an MiSeq platform (Illumina, United States) at the Shanghai Personal Biotechnology Co., Ltd. (Shanghai, China). All sequencing data were submitted to the NCBI database with accession number PRJNA645685. Sequencing data were processed using QIIME (version 1.8.0). In a few words, the original sequencing reads were sorted to obtain valid sequences. After filtering low-quality sequences, high-quality sequences were clustered into operational taxonomic units (OTUs) with a 97% sequence identity by UCLUST (Edgar, 2010). Then the most abundant sequence in each OTU was selected as the representative sequence. By comparing the representative sequences of OTUs with the template sequence of the Greengenes database (Desantis et al., 2006), the taxonomic information corresponding to each OTU was obtained. To minimize the difference in sequencing depth between samples, the average analysis of 100 evenly resampled OTU subsets under the 90% of the minimum sequencing depth was performed to generate an average, rounded dilution OTU table. Alpha-diversity values of each sample were assessed based on the observed OTUs, Chao1, and Shannon index. Beta-diversity measures dependent on weighted-UniFrac distance were calculated using mothur. LEfSe was conducted to identify bacterial taxa differentially represented between different groups at the genus or higher taxonomy level (Segata et al., 2011). PICRUSt analysis was used to predict the metagenome function of the microbiota (Langille et al., 2013).

### Statistical Analyses

The normal distribution of data was confirmed by the Shapiro-Wilk’s test before analysis. Variations in normally distributed semen traits, SCFAs, lactic acid, and markers associated with gut permeability and inflammation were examined by the Student-t test (Statistical Analysis System 9.4; Cary, NC, United States). Significance was reported when the *p*-value is less than 0.05. The dissimilarities of bacterial relative abundance between two groups and alpha diversity were evaluated using the Mann-Whitney U test. The predicted pathways from PICRUSt were established by using the Welch’s t-test in the STAMP software package (version 2.1.3). Correlations were investigated by using Spearman’s correlation in R 3.6.1 (the R Foundation) with the R Studio 1.2.1335 package and ggplot2 for the heat map. According to the false-discovery rate (FDR) procedure, the relevant results were corrected by FDR analysis, with *q* of <0.05.

## Results

### Semen Parameters of Duroc Boars

The semen parameters of the two group boars are given in Table 1. Though there is no significant difference in sperm concentration and total sperm number between the two groups, the low utilization rate group boars showed significantly lower semen volume (*p* < 0.05), functional sperm number (*p* < 0.01), sperm motility (*p* < 0.001), semen utilization rate (*p* < 0.001) and higher abnormal sperm rate (*p* < 0.001) compared with the high utilization rate group.

### Composition of Gut Microbiota in the Boars

**Figure 1:**
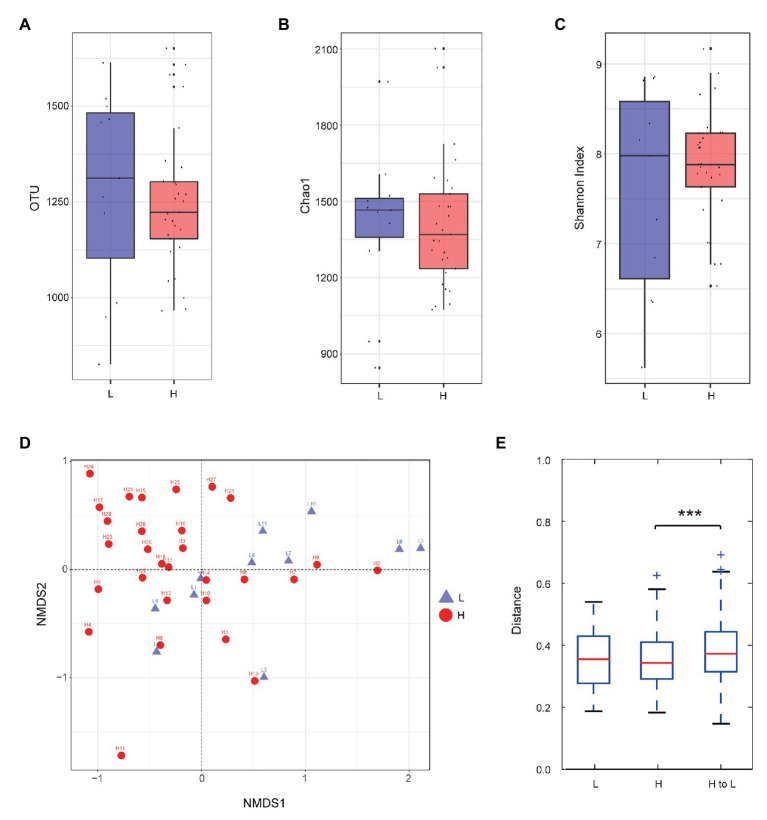
Alpha and beta diversity of gut microbiota. **(A**–**C)** Boxplots of observed operational taxonomic units (OTUs) **(A)**, Chao1 index **(B)**, and Shannon index **(C)** for the low semen utilization rate (L) and high semen utilization rate (H) groups. Red indicates the H group and blue indicates the L group. Boxes show the medians and the interquartile ranges (IQRs), the whiskers denote the lowest and highest values that were within 1.5 times the IQR from the first and third quartiles, and outliers are shown as individual points. **(D)** Nonmetric multidimensional scaling (NMDS) plot. The red circles indicate the H group, and the blue triangles indicate the L group. **(E)** Interindividual variations were determined by average weighted-UniFrac distances between individuals in the L and H group boars. Intraindividual variations were determined by distance-paired H and L group boars. ^***^p < 0.001.

**Figure 2:**
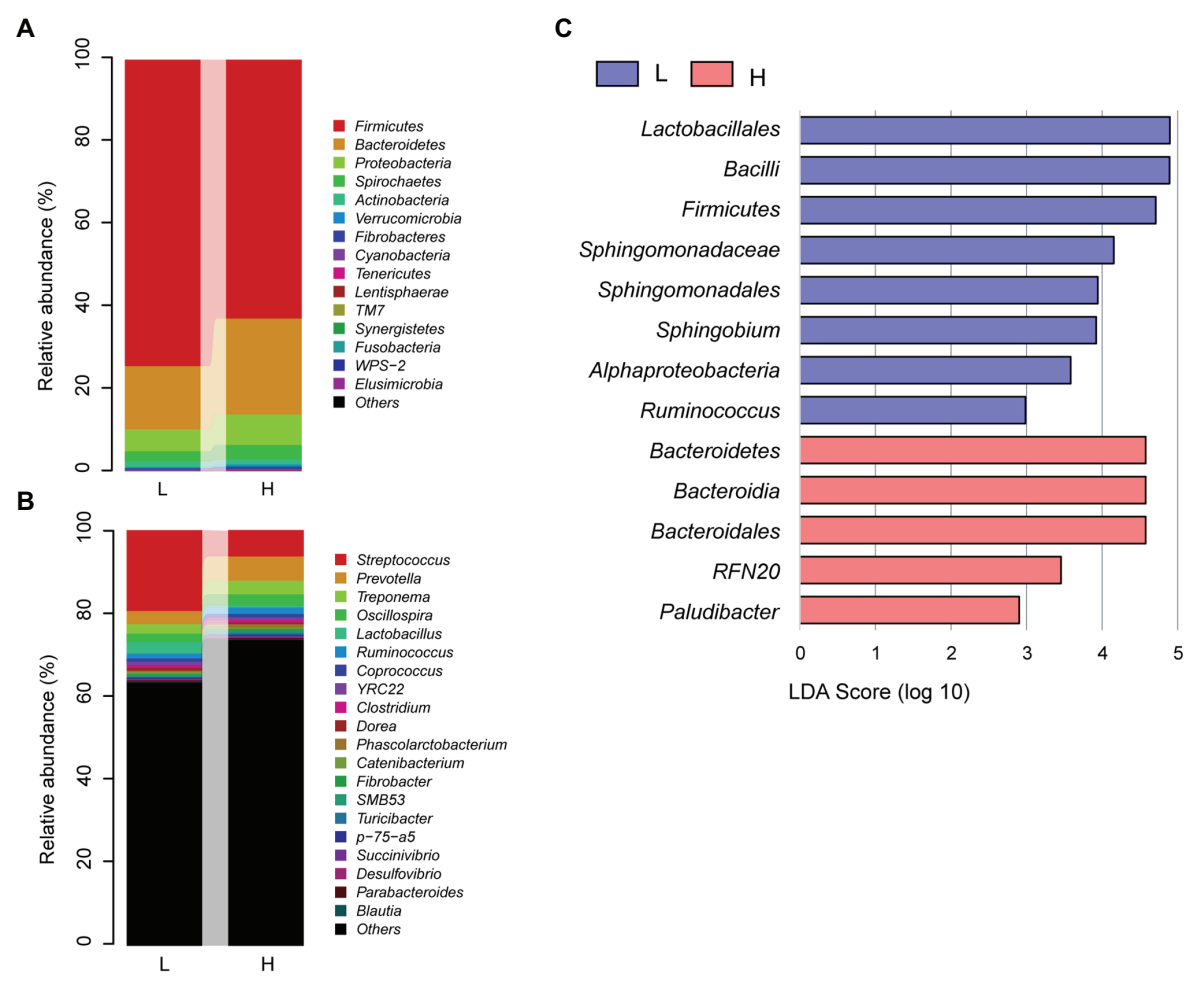
Microbial profiles at the phylum and genus level. **(A)** Barplot of relative abundance at the phylum level and **(B)** genus level for the L and H groups. The y-axis represents the relative abundance of each phylum for the two groups. **(C)** Linear discriminant analysis (LDA) score for discriminated genera in the L and H groups. The LDA score is calculated by LEfSe. The value suggests that it is increased in the two groups (*p* < 0.05, Wilcoxon rank-sum test, LDA > 2).

In order to study the composition of gut microbiota in different semen quality boars, 16S ribosomal RNA gene sequencing was used. A total of 1,407,378 high-quality sequencing reads were obtained from 40 samples, ranging from 28,112 to 46,845. Based on 97% species similarity, 50,406 OTUs were obtained from samples of the boars, which involved 7,265 different OTU classifications. The alpha and beta diversities were calculated. Alpha-diversity measures (observed OTUs, Chao1, and Shannon index) of the fecal bacterial community presented no significant differences between the two groups (Figures 1A–C). For beta diversity, the nonmetric multidimensional scaling (NMDS), based on weighted-UniFrac distance, exposed that the gut microbiota showed negligible segregation in the L and H group boars (Figure 1D). Although there is no clear visual separation of groups within the study boars in Figure 1D, the intergroup variation from H to L was higher than the interindividual variation of boars in group H (Figure 1E). The PERMANOVA analysis indicated small but significant differences in the gut microbiota between the L group and the H group (weighted-UniFrac distance, *R*^2^ = 0.083, *p* = 0.005). Furthermore, ANOSIM analysis based on weighted-UniFrac distance also confirmed that the microbial composition between the two groups was significantly different (*R* = 0.170, *p* = 0.036). As shown in Figure 2, the L and H group boars have different gut microflora composition at the phylum (Figure 2A) and the genus levels (Figure 2B). To identify the key genera in the gut microbiota of the L and H group boars, LDA was conducted to evaluate the effect size of each community that could effectively discriminate different groups (Figure 2C). LDA effect size revealed that in the genus level, two known genera (*Sphingobium* and [*Ruminococcus*]) were significantly enriched in the low utilization rate group boars, whereas two genera (*Paludibacter* and *RFN20*) were significantly enriched in the high utilization rate group boars (*p* < 0.05, Wilcoxon rank-sum test; log LDA > 2) (Figure 2C). The relative abundance of these four genera in each sample is shown in Supplementary Figure 1.

### Function Prediction of Gut Microbiota

**Figure 3:**
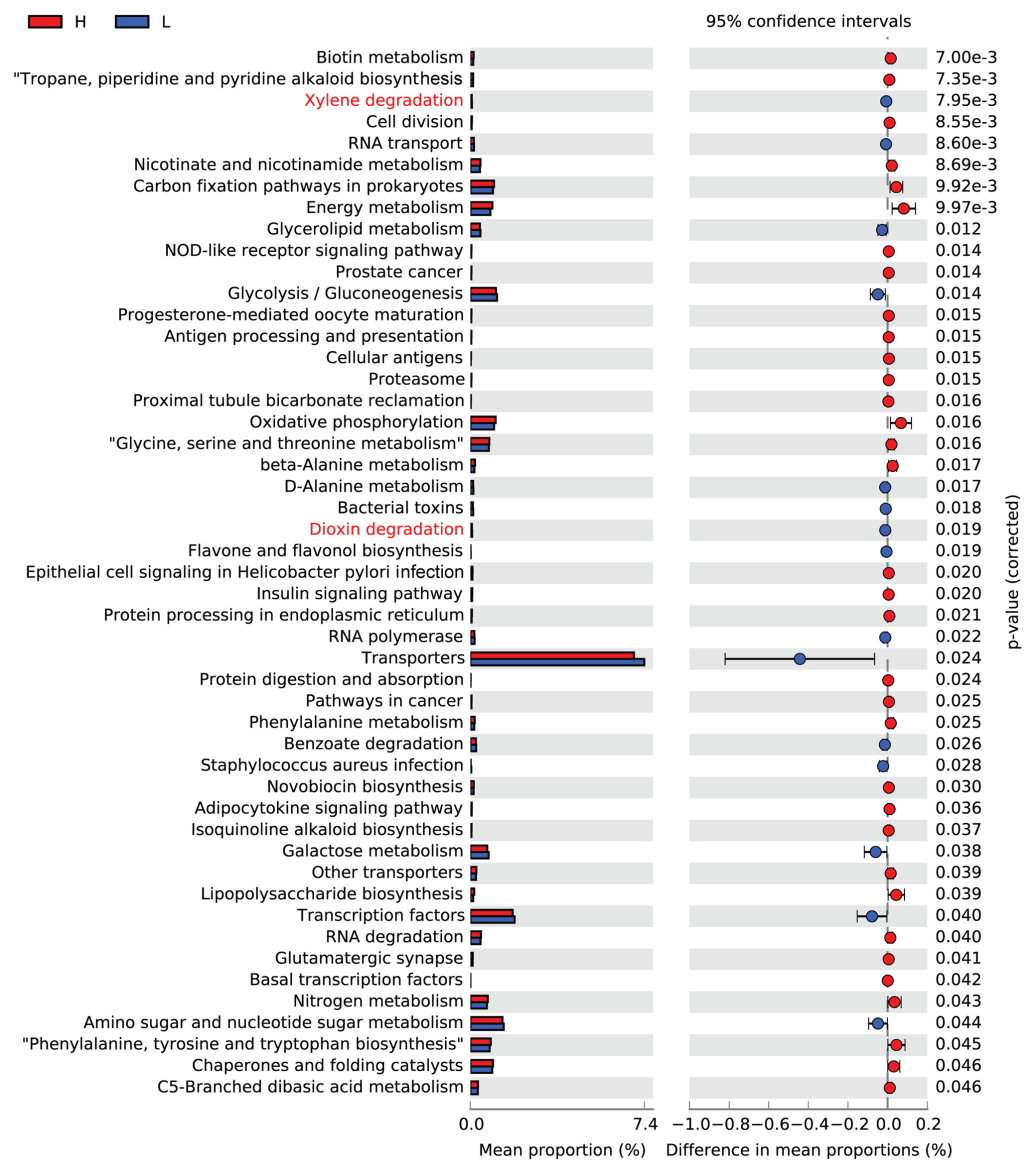
Analyses of pathways predicted by PICRUSt. The Mann-Whitney U test based on the PICRUSt data set revealed differentially enriched bacterial functions associated either with the L group (blue) or the H group (red).

Based on 16S rRNA sequences, PICRUSt analysis could be used to predict the potential functions of gut microflora. By comparing the KEGG database, it was found that the 49 metabolic pathways in the level 3 KEGG function classes are different between the L and H semen utilization rate group boars (Figure 3). The top three metabolic pathways with the largest differences between the two groups were related to biotin metabolism, tropane, piperidine and pyridine alkaloid biosynthesis, and xylene degradation. Genes for biotin metabolism, and tropane, piperidine, and pyridine alkaloid biosynthesis were significantly depleted in the L group in comparison with the H group. Nevertheless, xylene degradation functions were overrepresented in the L group when compared with the H group. It is worth mentioning that an increase in the function of dioxin degradation was also found in the L group.

### Analysis of Metabolites of Gut Microbiota

**Figure 4:**
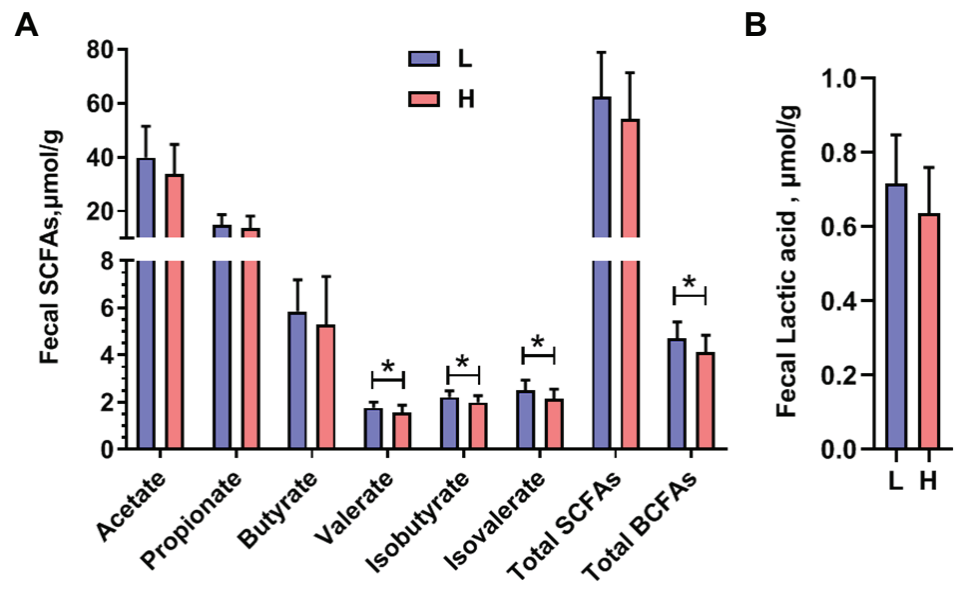
Content of **(A)** short-chain fatty acids (SCFA) and **(B)** lactic acid in the feces of different semen utilization rate group boars. *n* = 11 boars in the L group and 29 boars in the H group. ^*^p < 0.05.

To analyze the metabolites of the gut microbiota in boars of low or high semen utilization rate, we then focused on the fecal SCFAs and lactic acid. The results indicated that the concentration of fecal valerate, isobutyrate, isovalerate, and total acid BCFA from L group boars were higher (*p* < 0.05) than those from the H group boars, whereas no significant difference in the fecal level of acetate, propionate, butyrate, total SCFAs, and lactic acid was found between the L and H groups (Figure 4).

### Intestinal Permeability and Systemic Inflammation Levels of Boars

**Figure 5:**
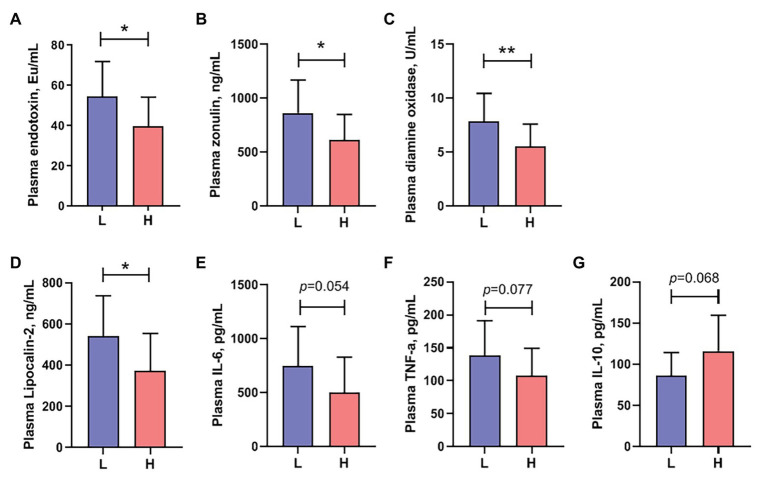
Plasma concentrations of **(A)** endotoxin, **(B)** zonulin, **(C)** diamine oxidase, **(D)** Lipocalin-2, **(E)** interleukin (IL)-6, **(F)** tumor necrosis factor (TNF)-α, and **(G)** IL-10 of different semen quality utilization rate group boars. Data are presented as mean ± SEM (*n* = 10 boars in the L group and 27 boars in the H group). ^*^p < 0.05, ^**^p < 0.01.

To determine the intestinal permeability of boars, we assessed endotoxin, zonulin, and diamine oxidase in the plasma. As shown in Figure 5, the plasma endotoxin level of boars with low semen utilization was significantly increased compared to boars with high semen utilization (Figure 5A). Similarly, the change trends of plasma zonulin and diamine oxidase concentration was consistent with endotoxin (Figures 5B,C). We further four parameters related to the immune activation and system inflammation. The results showed that the concentrations of plasma lipocalin-2 were significantly lower (*p* < 0.05) in the high semen utilization rate group boars; a similar trend was observed in the proinflammatory cytokines IL-6 (*p* = 0.054) and TNF-α (*p* = 0.077). However, different from the trend of proinflammatory cytokines, the concentration of anti-inflammatory mediator IL-10 (*p* = 0.068) in the plasma of boars with high semen utilization rate showed an increasing tendency (Figures 5D–G).

### Correlations Between Parameters Related to Boar Health

**Figure 6:**
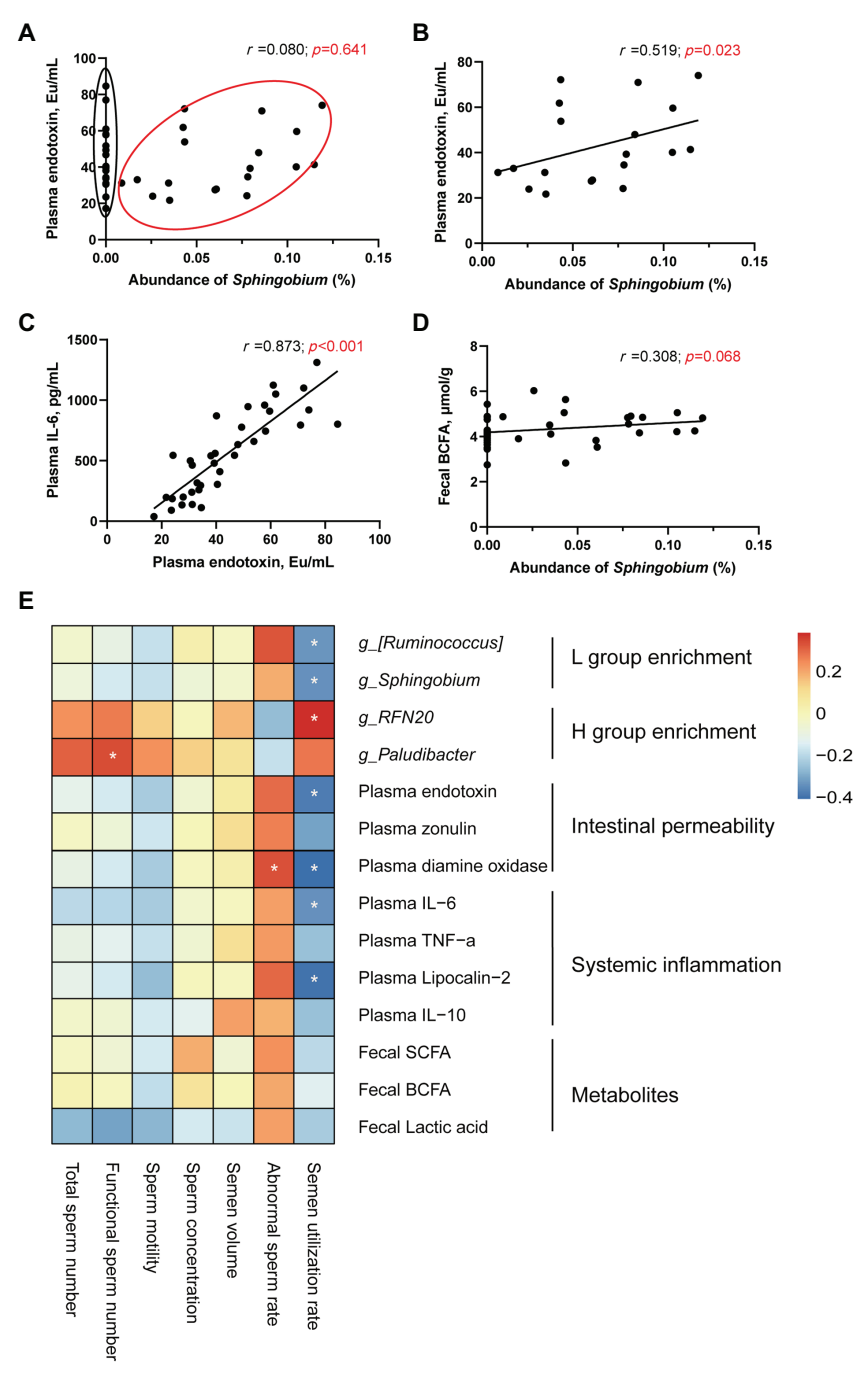
Spearman correlation analysis. **(A)** Correlation analysis between plasma endotoxin and the abundance of *Sphingobium* (*n* = 36). **(B)** Correlation analysis between plasma endotoxin and the abundance of *Sphingobium* (*n* = 19). **(C)** Correlation between plasma endotoxin and plasma IL-6. **(D)** Correlation between the abundance of *Sphingobium* and fecal BCFAs. **(E)** Heat map of the Spearman r correlations between the clinical status and semen parameters of boars. ^*^p < 0.05.

Spearman correlation analysis revealed that *Sphingobium* could be an important genus contributing to the intestinal permeability defect. When combining all the data together, the analysis showed a positive, and yet statistically not significant, association trend between plasma endotoxin and the abundance of *Sphingobium* (Figure 6A). However, after focusing on those boars’ samples with the abundance of *Sphingobium* > 0% (The relative abundance of *Sphingobium* in each sample is shown in Supplementary Figure 1), the results revealed a strong positive correlation (*n* = 19, *r* = 0.519, *p* = 0.023; Figure 6B), suggesting that high abundance of both *Sphingobium* or the other unidentified genera within *Proteobacteria* plays critical roles in regulating intestinal permeability. The plasma endotoxin concentration showed a positive correlation with plasma IL-6 (*n* = 36, *r* = 0.873, *p* < 0.001) (Figure 6C). Additionally, there was a weak positive correlation trend between the abundance of *Sphingobium* and fecal BCFAs (Figure 6D). Spearman correlation analysis was used to analyze the potential connections between clinical status and the semen parameters of boars (Figure 6E). The semen utilization rate was positively correlated with the genus *RFN20* (*p* < 0.05) but negatively correlated with the genera [*Ruminococcus*] (*p* < 0.05) and *Sphingobium* (*p* < 0.05), plasma endotoxin levels (*p* < 0.05), plasma diamine oxidase levels (*p* < 0.05), plasma IL-6 levels (*p* < 0.05), and plasma lipocalin-2 levels (*p* < 0.05). The abnormal sperm rate was positively correlated with the plasma diamine oxidase levels (*p* < 0.05). In addition, the functional sperm number was positively correlated with the genus *Paludibacter*.

## Discussion

The association between gut microbiota and host health has been widely studied. However, the effects of gut microbiota on semen quality were still less reported. In this study, we present evidence of such relationships in boars. The most significant findings of the present study are: (i) taxonomic and functional changes in the gut microbiome with boars in different semen utilization rate, and (ii) gut permeability dysfunction and altered immune status in low semen utilization rate boars. These findings suggested a novel relationship between gut microbiota and semen utilization rate in boar.

Using 16S ribosomal RNA gene sequencing analysis, we investigated both phylum and genus level changes in the boar gut microbiota. The results showed that different semen utilization rates of Duroc boars have diverse intestinal microbiota composition. The *Firmicutes* and *Bacteroidetes* phyla were the maximum abundant in total sequences, being similar to previous findings in the rectal microbiota of pigs (Wang et al., 2019). In addition, we also observed that *Sphingobium* and [*Ruminococcus*] were enriched in the L group, while *Paludibacter* and *RFN20* were enhanced in the H group boars. We hypothesized that analyzing the correlation of semen utilization rate and taxa abundance would be a better, more precise way to distinguish bacteria that had significant impacts on semen quality. Spearman correlation analysis showed that the genus *Sphingobium* and [*Ruminococcus*] were negatively correlated with the semen utilization rate, while the genus *RFN20* was positively correlated with it. *Sphingobium* can degrade many toxic organic pollutants, such as polycyclic aromatic compounds (Wittich et al., 2007). We found that the relative abundance of *Sphingobium* in the L group increased, and at the same time, the expression of metabolizing pollutants signaling pathways also increased. This means that boars in the L group may be exposed to more environmental pollutants, and pollutants may be a potential risk factor for the deterioration of boar semen quality (Bolden et al., 2015). [*Ruminococcus*], *Paludibacter* and *RFN20* are capable of degrading dietary fiber and fermenting complex carbohydrates (Ze et al., 2012; Fernandes et al., 2014; Cui et al., 2019). SCFAs are mainly fermented products that escape the absorption of carbohydrates and proteins in the small intestine during digestion (Rios-Covian et al., 2016). SCFAs, especially butyrate, is beneficial in regulating the gut barrier (Huang et al., 2015) and also protects against inflammation (Flint et al., 2012). However, in this study, alterations in gut permeability and inflammatory status in the L group boars may not be related to SCFAs because the data showed that there was no significant difference in fecal concentrations of SCFAs between the L and H groups. However, levels of fecal BCFAs in the L group were higher than those of the H group boars. BCFAs can be considered as markers of proteolysis in the colon. It is believed that protein fermentation can result in toxic or potentially toxic metabolites such as ammonia, BCFAs (e.g., isobutyrate and isovalerate), and phenolic compounds (Macfarlane and Macfarlane, 2012; Nyangale et al., 2012). The increase in BCFA content in the L group may be owing to the enrichment of *Proteobacteria*. Bacteria of *Proteobacteria* are mainly responsible for the use of amino acids (Booijink, 2009), and in our study, we found a weak positive correlation tendency between the abundance of *Sphingobium* and fecal BCFA.

Likewise, we found an increased abundance of Gram-negative *Proteobacteria* (*Alphaproteobacteria*, *Sphingomonadales*, *Sphingomonadaceae*, and *Sphingobium*) in the L group boars. *Proteobacteria* is a minor component in the balanced gut-associated microbial community (Eckburg et al., 2005). Expansive of *Proteobacteria* is observed during conditions of low-level intestinal inflammation, including irritable bowel syndrome and metabolic syndrome (Morgan et al., 2012). Numerous studies have shown that the abnormal expansion of *Proteobacteria* is a potential diagnostic microbial characteristic of gut microbiota imbalance and epithelial dysfunction (Shin et al., 2015; Litvak et al., 2017). Then, we evaluated boar intestinal permeability and systemic inflammatory response. We found that the contents of the three indicators of intestinal permeability of boars with low semen utilization rates were increased. Furthermore, consistent with what was mentioned before, we found a positive correlation between plasma endotoxin and the abundance of genus *Sphingobium.*

Circulating LPS activates the immune system *via* toll-like receptors, which result in proinflammatory cytokines like IL-6 or TNF-α and acute-phase protein production (Kimball et al., 2003; de La Serre et al., 2010; Rakhshandeh and De Lange, 2012). In this study, we found that the plasma endotoxin concentration of boars with low semen utilization was significantly increased, accompanied by an increase in the level of systemic inflammation. Spearman analysis showed that the concentration of plasma endotoxin was positively correlated with IL-6. The above findings are consistent with previous studies. Proinflammatory conditions may lead to increased ROS formation, and sperm membrane lipid peroxidation decreased motility and increased DNA damage (Urata et al., 2001; Kasturi et al., 2008). Besides, male hypogonadism may be associated with the chronic inflammation that inhibits testosterone synthesis (Kalyani and Dobs, 2007). Both LPS and proinflammatory cytokines can affect the production of sex steroids. Nevertheless, decreased testosterone is associated with many adverse reactions, including reduced sperm production, libido, and sexual function (Matsumoto, 2002; Bassil and Morley, 2010).

Additionally, we also explored the function of the gut microbiota of boars with different semen utilization rates. Among these functional pathways, we noticed that xylene degradation and dioxin degradation pathways are increasingly expressed in L group boars. This may be related to the increased abundance of *Sphingobium*. Members of this genus are mostly found in saprophytic soils and water, and many isolates are involved in the biodegradation of many toxic organic pollutants such as nonylphenol (Ushiba et al., 2003), phenanthrene (Prakash and Lal, 2006), and polycyclic aromatic compounds (Wittich et al., 2007). Environmental pollutants of xylene and dioxins have carcinogenic effects and reproductive toxicity on animals (Bolden et al., 2015; Johnson et al., 2020). These indicate that the boar semen quality is affected by environmental pollutants. The above analysis further confirms that the gut microbial structure and microbial metabolism are shaped in boars with low semen utilization.

Our findings supported the changes in gut microbiota composition and function of the different semen utilization rate boars. However, the reasons for these changes are still unclear and needs further investigation. Additionally, the semen utilization rate is affected by many factors. Through this study, we were unable to determine the specific mechanism by which gut microbiota changed semen parameters. Nonetheless, given the overall importance of gut microbiota to male fertility, there is no doubt that further research is needed to clarify these potential mechanisms. Our research exhibited that the structure and function of the gut microbiota of boars with different semen utilization rates have changed significantly. Specifically, the accumulation of *Sphingobium* and other *Proteobacteria* bacteria in the gut of the L group boars may contribute to the increase in plasma endotoxins, which in turn will cause systemic inflammation and reduce the quality of boar semen. This study suggested the potential correlation of the semen utilization rate of boars with gut microbiota, intestinal permeability, and inflammation status stress. This investigation provided some novel insights into the differences between the gut microbiota and microbial function of boars with different semen utilization rates. We reported first the vital effects of gut microbiota on boar semen quality and provided a new perspective for understanding male fertility.

## Data Availability Statement

All sequencing data were uploaded to the NCBI database with accession number PRJNA645685.

## Ethics Statement

The animal study was reviewed and approved by Institutional Animal Care and Use Committee of Huazhong Agricultural University (permit number HZAUSW-2018-014). Written informed consent was obtained from the owners for the participation of their animals in this study.

## Author Contributions

JP conceived and designed the experiments, and wrote and revised the manuscript. SJ conceived and designed the experiments, and revised the manuscript. LG performed the experiments, analyzed the data, and wrote part of the manuscript. YW performed the experiments and took part in the data analysis. CW and HW analyzed the data. JT and HS collected the samples. All authors contributed to the article and approved the submitted version.

### Conflict of Interest

JT and HS were employed by the company YangXiang Joint Stock.

The remaining authors declare that the research was conducted in the absence of any commercial or financial relationships that could be construed as a potential conflict of interest.
