# Supplementary material for: Gut Microbiological Disorders Reduce Semen Utilization Rate in Duroc Boars
Source: Front Microbiol. 2020 Oct 8;11:581926. doi: 10.3389/fmicb.2020.581926 (PMC7578402; doi:10.3389/fmicb.2020.581926)
Supplement: Supplementary file 1 [file Data_Sheet_1.pdf]

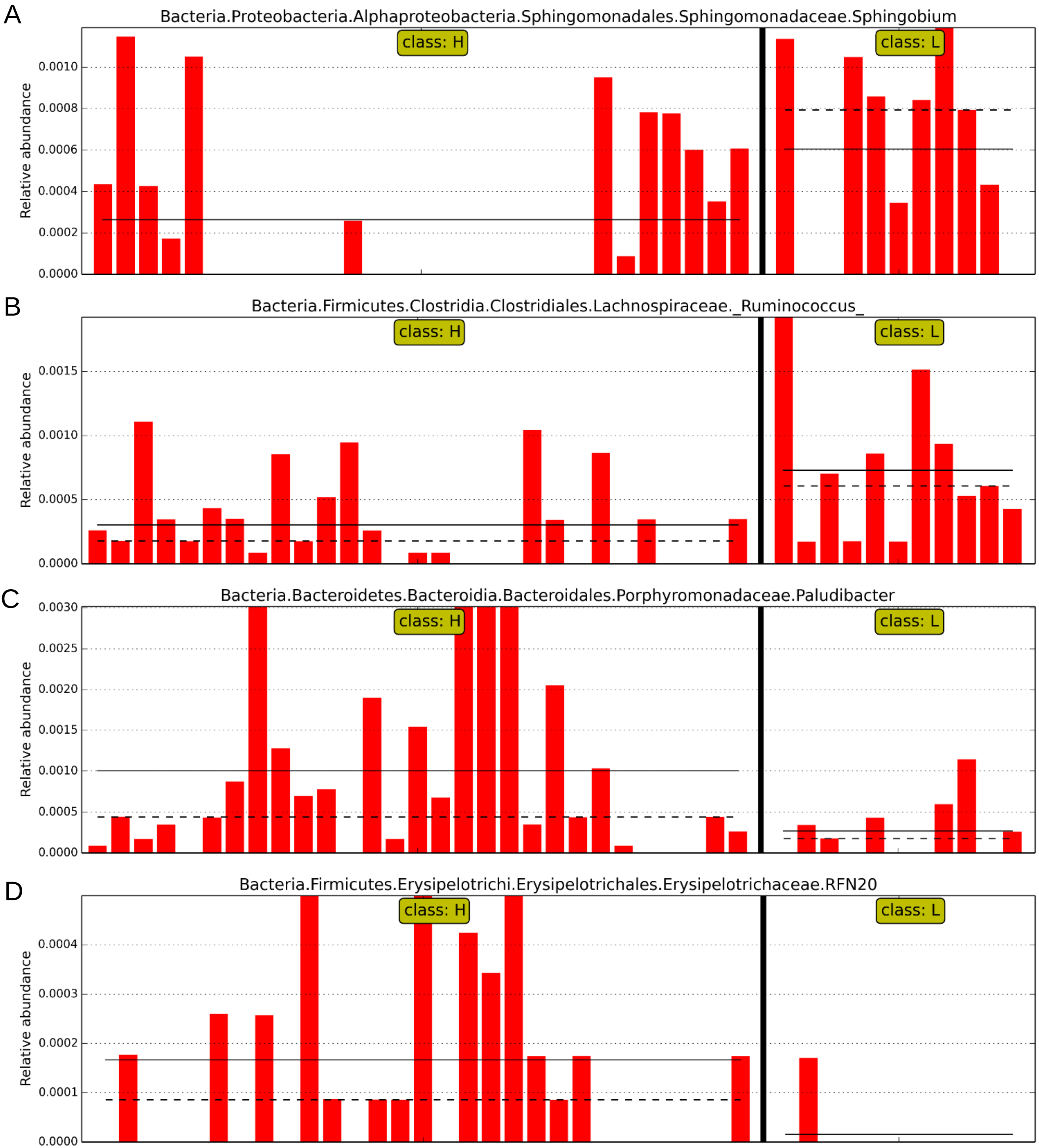

Supplementary Figure 1. The relative abundance distribution of taxa with significant differences in different groups. (A) *Sphingobium* , (B) *Ruminococcus*, (C) *Paludibacter*, (D) *RFN20*. The solid and dashed lines indicate the average and median relative abundance of the genus in each group.
